# Supplementary material for: Selective Chemical Labeling and Sequencing of 5-Hydroxymethylcytosine in DNA at Single-Base Resolution
Source: Front Genet. 2021 Nov 17;12:749211. doi: 10.3389/fgene.2021.749211 (PMC8635956; doi:10.3389/fgene.2021.749211)
Supplement: Supplementary file 1 [file Table3.doc]

Table S3. Primer List for CH Sites Validation

| Type | chr | C position | Forward Primer | Reverse Primer | | Ct  (BGT) | | Ct  (BGT+Aba) |
| --- | --- | --- | --- | --- | --- | --- | --- | --- |
| CHXX | 5 | 147915464 | ACCAAGACCTCACTGCCATC | CCCTGCAGTCTGGCTTATTC | 23.14 | | 23.65 | |
| CHXX | 1 | 67448459 | CCACTGCATTCACACCATTT | AAAGCTTTGTCAGGAAGCCA | 22.87 | | 23.45 | |
| CHCH | 1 | 59220769 | TGCCAAAGGGTATTGGGTAA | TAGAGCCCAGCATGGAAAAC | 22.82 | | 23.57 | |
| NC | 1 | 31116274 | GGCTGGTGTTCAGGATCTGT | ATTGCTAAGGCAGCAGGAAG | 23.53 | | 23.47 | |
| NC | 19 | 59988426 | CCGTGCATTCACAGAGTCTTC | CAGATGTTCTTGGGGGAAAA | 23.21 | | 23.15 | |

Aba-seq is a method using DNA-modification dependent restriction endonuclease AbaSI coupled with sequencing, which enables sensitive

detection of 5-hydroxymethylcytosine (5hmC) at low-occupancy regions.

NC: Negative controls, NC are sequence with CH sites in the AbaSI recognition sequence context but are not treated with AbaSI.

Ct (BGT) and Ct (BGT+Aba) is real-time PCR Ct value for specific treated genomic DNA. Ct (BGT): gDNA was treated with T4-β-glucosyltransferase without AbaSI digestion; Ct (BGT+Aba) : gDNA was first glucosylated by T4-β-glucosyltransferase and then was digested with AbaSI. If the specific region (cytosine) is hydroxymethylated, it will be digested by AbaSI, so Ct (BGT+Aba) would be higher than Ct (BGT).

References

Sun Z, Terragni J, Borgaro JG, Liu Y, et al. 2013 High-resolution enzymatic mapping of genomic 5-hydroxymethylcytosine in mouse embryonic stem cells. Cell Rep. 3(2): 567-76.
