# Supplementary material for: Selective Chemical Labeling and Sequencing of 5-Hydroxymethylcytosine in DNA at Single-Base Resolution
Source: Front Genet. 2021 Nov 17;12:749211. doi: 10.3389/fgene.2021.749211 (PMC8635956; doi:10.3389/fgene.2021.749211)
Supplement: Supplementary file 2 [file Table1.DOC]

**Table S1 comparative analysis**

| Method | seq-num | trimmomatic | bam-reads | bowtie2_0times | bowtie2_1times | bowtie2_2times |
| --- | --- | --- | --- | --- | --- | --- |
| nano-seal | SEQ19585 | 0.979 | 32840003 | 0.3021 | 0.5952 | 0.1027 |
| nano-seal | SEQ19590 | 0.9772 | 37038581 | 0.2601 | 0.6298 | 0.11 |
| ace-seq | SEQ19586 | 0.9876 | 34104219 | 0.2529 | 0.6746 | 0.0725 |
| ace-seq | SEQ19587 | 0.9875 | 36030896 | 0.2455 | 0.6839 | 0.0706 |
| ace-pulldown | SEQ19588 | 0.8946 | 34705347 | 0.3574 | 0.5883 | 0.0543 |
| ace-pulldown | SEQ19589 | 0.9198 | 30832711 | 0.3114 | 0.6294 | 0.0592 |
