# Supplementary material for: Selective Chemical Labeling and Sequencing of 5-Hydroxymethylcytosine in DNA at Single-Base Resolution
Source: Front Genet. 2021 Nov 17;12:749211. doi: 10.3389/fgene.2021.749211 (PMC8635956; doi:10.3389/fgene.2021.749211)
Supplement: Supplementary file 3 [file Table2.DOC]

Table S2 Statistics results of 5hmC sites

| Method | seq_num | total_lines | total_strings | total_C | hmC_CpG | hmC_CHG | hmC_CHH | C2T_CpG | C2T_CHG | C2T_CHH |
| --- | --- | --- | --- | --- | --- | --- | --- | --- | --- | --- |
| ace-seq | SEQ19586 | 18191578 | 36383156 | 756963288 | 6342475 | 20163184 | 62652700 | 27525310 | 142500601 | 497779018 |
| ace-seq | SEQ19587 | 18824449 | 37648898 | 812058698 | 1183130 | 3235097 | 11574624 | 32824248 | 170305710 | 592935889 |
| ace-  pulldown | SEQ19588 | 12680153 | 25360306 | 614206950 | 5427793 | 1418759 | 4081655 | 41829555 | 144165807 | 417283381 |
| ace-  pulldown | SEQ19589 | 12522156 | 25044312 | 598297204 | 5818168 | 2586082 | 7293592 | 41661802 | 140467042 | 400470518 |
